# Decoding the immune landscape: a comprehensive analysis of immune-associated biomarkers in cervical carcinoma and their implications for immunotherapy strategies

**DOI:** 10.3389/fgene.2024.1340569

**Published:** 2024-06-12

**Authors:** Le Wang, Huatian Liu, Yue Feng, Xueting Liu, Yuan Wang, Yujie Liu, Hao Li, Yunyan Zhang

**Affiliations:** ^1^ Department of Gynecological Radiotherapy, Harbin Medical University Cancer Hospital, Harbin, China; ^2^ Department of Anesthesiology, The First Affiliated Hospital of Harbin Medical University, Harbin, China; ^3^ Department of Gynecological Oncology, Zhejiang Cancer Hospital, Hangzhou Institute of Medicine (HIM), Chinese Academy of Sciences, Hangzhou, China; ^4^ Harbin Medical University Cancer Hospital, Harbin, China

**Keywords:** CESC, TIME, ICI therapy, IRGPI, therapeutic target

## Abstract

**Background and aims:**

Cervical cancer, a prevalent gynecological malignant tumor, poses a significant threat to women’s health and lives. Immune checkpoint inhibitor (ICI) therapy has emerged as a promising avenue for treating cervical cancer. For patients with persistent or recurrent metastatic cervical cancer, If the sequence of dead receptor ligand-1 (PD-L1) is positive, ICI show significant clinical efficacy. PD-L1 expression serves as a valuable biomarker for assessing ICI therapeutic efficacy. However, the complex tumor immune microenvironment (TIME), encompassing immune cell composition and tumor-infiltrating lymphocyte (TIL) status, also exerts a profound influence on tumor immunity and prognosis. Given the remarkable strides made by ICI treatments in improving the survival rates of cervical cancer patients, it becomes essential to identify a comprehensive biomarker that integrates various TIME aspects to enhance the effectiveness of ICI treatment. Therefore, the quest for biomarkers linked to multiple facets of TIME in cervical cancer is a vital pursuit.

**Methods:**

In this study, we have developed an Immune-Associated Gene Prognostic Index (IRGPI) with remarkable prognostic value specifically for cervical squamous cell carcinoma and endocervical adenocarcinoma (CESC). The Cancer Genome Atlas CESC dataset (*n* = 305) was meticulously analyzed to pinpoint key immune-related genes via weighted gene co-expression network analysis and differential gene expression assays. Subsequently, we employed Cox regression analysis to construct the IRGPI. Furthermore, the composition of immune cells and TIL status were examined using CIBERSORT and TIDE. Tumor expression of Epigen, LCN10, and P73 were determined with immunohistochemistry.

**Results:**

The resulting IRGPI, composed of EPGN, LCN10, and TP73 genes, displayed a strong negative correlation with patient survival. The discovery was validated with a patient cohort from our hospital. The IRGPI not only predicts the composition of immune cell subtypes such as Macrophages M1, NK cells, Mast cells, Plasma cells, Neutrophils, Dendritic cells, T cells CD8, and T cells CD4 within CESC, but also indicates TIL exclusion, dysfunction, and PD-1 and PD-L1 expression. Therefore, the IRGPI emerges as a promising biomarker not only for prognostic assessment but also for characterizing multiple immune features in CESC. Additionally, our results underscored the significant associations between the IRGPI and immune cell composition, TIL exclusion, and dysfunction, along with PD-1 and PD-L1 expression in the TIME.

**Conclusion:**

Consequently, the IRGPI stands out as a biomarker intimately connected to both the survival and TIME status of CESC patients, offering potential insights into immunotherapy strategies for CESC.

## 1 Introduction

Globally, cervical carcinoma ranks as the fourth most common malignancy and the fourth-highest cause of cancer-related death in women (Ding et al., 2020; Liu et al., 2020). Despite significant advancements in cervical screening and a variety of treatment modalities, including radiotherapy, chemotherapy, and surgery (Jemal et al., 2011; Torre et al., 2015; Bray et al., 2018), the incidence and mortality rates of cervical cancer have continued to rise annually worldwide. Patients with advanced cervical cancer may develop resistance to radiotherapy and chemotherapy, leaving limited treatment options upon recurrence and metastasis. Consequently, there is an imperative need to explore novel prognostic markers and therapeutic targets that could provide essential prognostic information to guide the management of metastatic and recurrent CESC.

In recent years, immunotherapy has achieved remarkable progress in clinical anticancer therapy, emerging as a new frontline treatment for recurrent cervical cancer (Polk et al., 2018). Notably, clinical trials such as KEYNOTE-158 and KEYNOTE-826 have demonstrated the effectiveness of immunotherapy in improving survival rates for CESC patients (Chung et al., 2019; Monk et al., 2023). While other cancers have employed several standards to assess the potential benefits of anti-PD-1/PD-L1 therapy, cervical cancer faces unique challenges. For instance, mismatch repair defects and high tumor mutation burden are relatively rare in cervical cancer (Le et al., 2017; Friedman et al., 2023), making PD-L1 expression the primary biomarker for ICI treatment. However, limitations in PD-L1 detection methods and its variability among patients hinder its reliability (Rimm et al., 2017). Moreover, a subset of PD-L1-negative patients still responds positively to ICI treatment (Ribas and Siwen, 2016). Although immune checkpoint inhibitors were expected to exhibit great potential in CESC patients, the clinical outcomes and prognosis have been less than satisfactory, with fewer than 20% of patients achieving partial or complete responses, even among those with PD-L1-positive tumors (Frenel et al., 2017; Chung et al., 2019).

The tumor microenvironment (TME) constitutes a complex ecosystem comprising various cell types, including fibroblasts, vascular endothelial cells, and their secreted products such as cytokines and chemokines, which play pivotal roles in tumor initiation and progression (Joyce and Pollard, 2009; de Vos van Steenwijk et al., 2013; Quail and Joyce, 2013; Riaz et al., 2016; Cabel et al., 2017; Liu et al., 2019; Okonogi et al., 2019; Pignata et al., 2019). Tumor-infiltrating immune cells (TICs) have a direct bearing on the response to immunotherapy. Therefore, in-depth analysis of the composition and characteristics of TICs in CESC and their correlation with infiltration patterns and prognosis becomes essential for a nuanced understanding of the complex antitumor response and effective guidance for immunotherapy. Thus, a dependable prognostic feature is indispensable to make precise personalized decisions regarding ICI treatment, assess prognosis, and predict immunotherapy responsiveness in CESC patients.

Promisingly, a prognostic signature based on immune-related genes (IRGs) has shown tremendous potential in predicting prognosis and immunotherapy responsiveness across various cancers, although such models have been less explored in the context of CESC. In this study, we have formulated a three-gene signature (EPGN, LCN10, and TP73) as an IRGPI to forecast CESC prognosis and immune characteristics. We systematically analyzed immune-related genes within CESC transcriptome data and identified hub genes linked to patient prognosis through weighted gene co-expression network analysis (WGCNA). The IRGPI’s prognostic value was subsequently validated, and its associations with tumor-immune cell profiles, TIL status, and PD1/PD-L1 immune checkpoints were further elucidated. Our results unequivocally affirm that the IRGPI is an exceptional biomarker for forecasting both prognosis and TIME status in CESC. To the best of our knowledge, this comprehensive multi-gene model assessing survival in cervical cancer, significantly associated with the TME, is unparalleled in the field. Ultimately, our IRGPI-based prognostic signature not only serves as a reliable tool for survival prediction but also efficiently predicts the clinical response of ICIs for CESC patients. This achievement holds the potential to advance personalized consultation for immunotherapy in the foreseeable future.

## 2 Materials and methods

### 2.1 Data collection and immune genes sources

RNA-seq data from cervical cancer patients, including 305 cancer samples and 3 adjacent normal tissue samples, were obtained from the Cancer Genome Atlas (TCGA) database. Clinical data such as survival time, status, age, TNM, and stage were sourced from UCSC Xena (accessed on 11 October 2022). Immune-related genes were retrieved from the ImmPort database (ImmPort: 1793) and InnateDB (InnateDB: 1226), leading to a total of 2660 immune genes after duplicates were removed. Of these, 1855 genes were retained for further analysis after genes with expression loss in more than 70% of samples in the pretreatment data were excluded.

### 2.2 Weighted gene coexpression network analysis (WGCNA)

WGCNA is a robust tool for identifying clusters (modules) of highly interconnected genes. We employed WGCNA to identify co-expression modules of immune-related genes from ImmPort and InnateDB. This involved constructing a co-expression similarity matrix based on Pearson correlation coefficients between gene pairs. Utilizing the scale-free topology criterion (R2 = 0.9) and a soft threshold (β = 3), we established five modules. Each module, except the gray module, represented a set of genes with high patient-to-patient similarity. Genes not assigned to any module were placed in the gray module. We used topological overlap matrices (TOM) for visualizing gene-gene connectivity and carried out GO and KEGG pathway analyses for genes in each module (except the gray module) using R’s clusterProfiler package to identify enriched pathways. Correlations between the four modules obtained through WGCNA and sample features (cervical cancer status) were computed, revealing that genes in the yellow and brown modules were significantly correlated with cervical cancer (*p* values of 1e-15 and 0.004, respectively), indicating their potential as cervical cancer targets. Genes from the yellow and brown modules were selected for subsequent analysis.

### 2.3 Identification of differentially expressed genes

Differential gene expression analysis was performed using RNA-seq data from cervical cancer patients (tumor and three normal samples). Genes with |log2FoldChange| ≥ 1 and an adjusted *p*-value (Benjamini-Hochberg method) < 0.05 were considered significantly differentially expressed between the samples.

### 2.4 Construction and validation of immune-related gene prognostic index (IRGPI)

Univariate Cox regression analysis identified immune-related differentially expressed genes (DEGs) significantly correlated with overall survival in cervical cancer patients (*p*-value <0.05). Multi-factor Cox regression and stepwise regression analysis were employed to construct the IRGPI model. The final optimal regression model’s coefficients were calculated. The IRGPI score for each cancer sample was calculated as follows: gene one expression level * coef1 + gene 2 expression level * coef2 + … + gene N expression level * coefN. Patients in the TCGA dataset were divided into high and low IRGPI groups based on IRGPI scores. The Kaplan-Meier (KM) survival curve assessed the IRGPI model, and the log-rank test further confirmed its performance. This prognostic model was also validated in a separate cohort by calculating the area under the ROC curve (AUC) at 250, 500, and 750 days. Multivariate Cox regression analysis incorporated significant clinicopathological features (age, TNM, and stage) and IRGPI scores for cervical cancer patients.

### 2.5 Identification of molecular characteristics between different IRGPI subgroups

Differential expression analysis was conducted between groups with high (*n* = 153) and low (*n* = 153) IRGPI scores. Enrichment analysis and gene set enrichment analysis (GSEA) were performed on DEGs from high and low IRGPI groups to identify relevant signaling pathways.

### 2.6 Assessment of immune cell infiltration

CIBERSORT was used to evaluate the infiltration levels of immune cells in each sample. The Wilcoxon test compared immune cell distribution among different subgroups, and Spearman correlation was used to calculate immune cell correlations.

### 2.7 GO and KEGG analysis of immune-associated DEGs

Immune-associated DEGs were identified, and R’s clusterProfiler package was used to perform GO and KEGG analyses to identify significantly enriched biological processes (BP), cellular components (CC), molecular functions (MF), and pathways (corrected *p*-values <0.05).

### 2.8 TIDE analysis

TIDE was employed to assess individual responses to immunotherapy. The Wilcoxon rank sum test was used to calculate differences in TIDE scores among IRGPI groups (*p* < 0.05). Spearman correlation analyzed correlations between prognostic markers (IRGPI, EPGN, LCN10, TP73) and PD-L1/PD1 expression.

### 2.9 Specimen and clinical data

Specimen and Clinical Data Formalinfixed, paraffin-embedding (FFPE) specimens were collected from the Harbin Medical University Cancer Hospital. Tissues were collected from 59 CESC patients who underwent treatment of cervival cancer between 2012 and 2016 in Harbin Medical University Cancer Hospital. The study was approved by the Ethics Committee of Harbin Medical University Cancer Hospital. Follow-up time ranged from 1 to 122 months.

### 2.10 Immunohistochemistry

Tissue sections were dewaxed in xylene and hydrated gradually through graded alcohol. EDTA buffer was used for antigen retrieval. The endogenous peroxidase activity was blocked, and then the sections were incubated with the primary anti-Epigen (1:100; PA5-47985,ThermoFisher),anti-LCN10(1:200; PA5-63207,ThermoFisher), or anti-P73 (1:100; PA5-35368,ThermoFisher) overnight at 4°C. Then the secondary antibody and a DAB kit (34,002, Thermo Scientific™) were applied to the sections. The tissue sections were evaluated by two pathologists who were unaware of the patient’s clinical information. For Epigen, LCN10, and P73 IHC, the scores were assigned from 0 to 3 based on the staining intensity level (no staining, light brown, brown, and tan). The staining extent was graded from 0 to 4 for the percentage of positive cells (0%–5%, 5%–25%, 26%–50%, 51%–75%, and 76%–100%). The product of staining intensity and extent scores was used as the IHC score, with a range of 0–12. Scores 0–4 were assigned as negative, and scores 5–12 were assigned as positive.

### 2.11 Clinical data validation

Clinical and transcript data from a cohort of patients treated with IMvigor210 for metastatic urothelial carcinoma were retrieved using the “IMvigor210CoreBiologies” package (*n* = 348).

### 2.12 Statistical analysis

All statistical analysis was performed with R language. The Cox regression analysis was used to determine hazard ratios (HRs) and 95% confidential intervals (CIs) for univariable and multivariable analyses. Progression-free survival (PFS) was defined as the time between the initiation of diagnosis and the date of the first evidence of tumor progression. We used Kaplan–Meier plots and log-rank tests to calculate the differences in PFS among different subgroups. The Pearson correlation analysis was used to calculate the correlation of two variables. *p* < 0.05 was defined as a statistical significance.

## 3 Results

### 3.1 Identification of gene co-expression networks of immune-related genes in CESC samples

**FIGURE 1:**
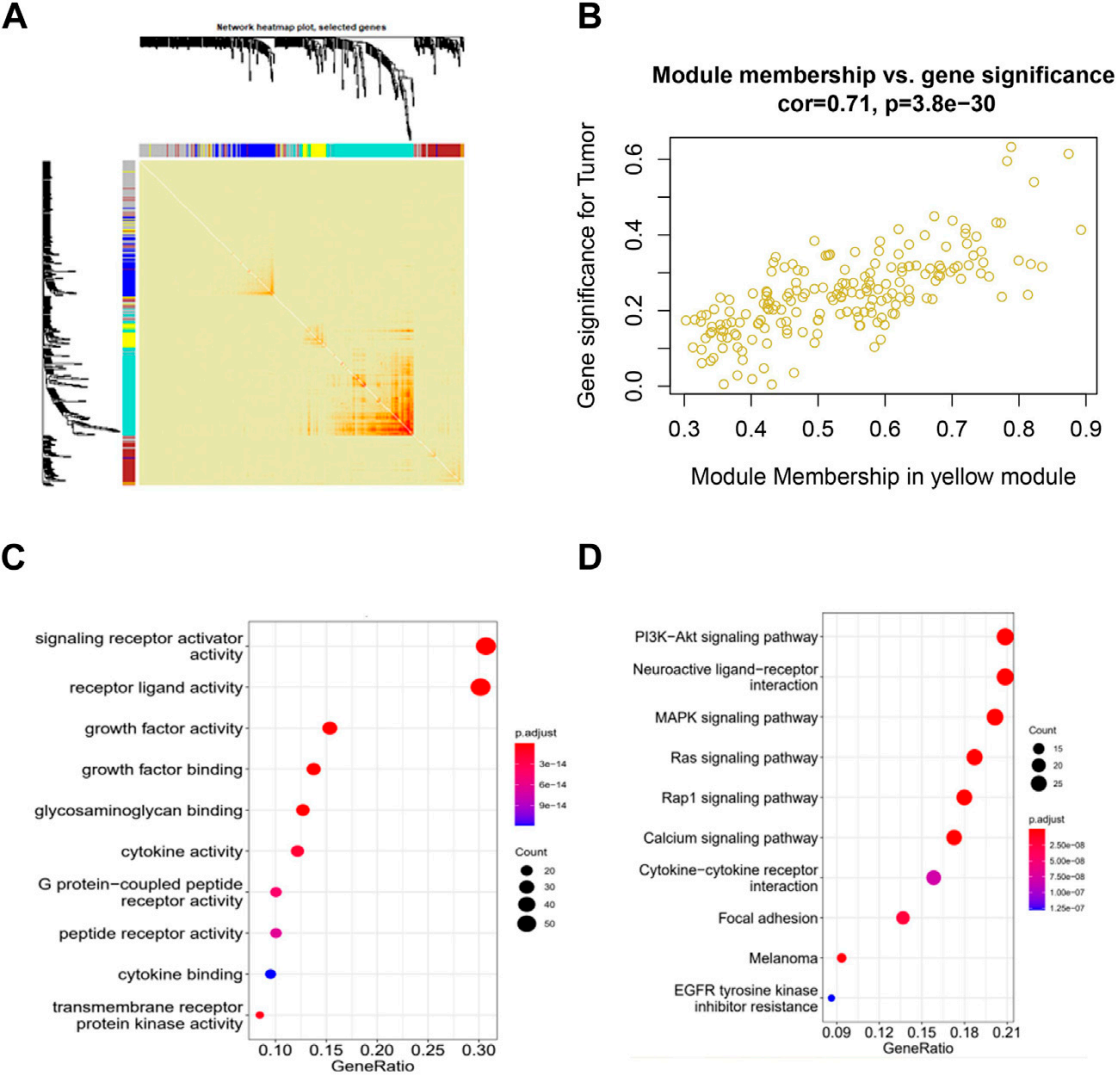
Identification of co-expressed immune-related genes associated with CESC using WGCNA. **(A)** WGCNA analysis topological overlap matrix heatmap; **(B)** Correlation between genes in the yellow module and CESC; **(C)** Enrichment of GO genes in the co-expression network yellow module; **(D)** Enrichment of KEGG genes in the co-expression network yellow module.

To identify immune-related hub genes, we conducted a WGCNA analysis on immune genes gathered from the ImmPort and InnateDB databases. The co-expression modules of immune-related genes were determined using the WGCNA method. The results were visualized in different colored modules. Five modules were identified using a soft threshold of 3. A total of 1855 genes were assigned to these five modules, consisting of 298 in the blue module, 296 in the brown module, 529 in the turquoise module, 191 in the yellow module, and 541 in the gray module (Figures 1A; Supplementary Figure S1B, S2C).

When we clustered the samples, we observed that the expression profiles of cervical cancer samples corresponded well to the sample classification, which has little relationship with WGCNA itself (Supplementary Figure S2A). Our WGCNA analysis revealed that the genes in the yellow and brown modules showed significant correlations with cervical cancer, with *p*-values of 1e-15 and 0.004, respectively. This suggests that the genes in the yellow and brown modules may hold potential as targets for cervical cancer (Supplementary Figure S2B). Analyzing within the modules, the genes in the yellow and brown modules exhibited strong correlations with CESC, with correlation coefficients of 0.71 and 0.6, and respective *p*-values of 3.8e-30 and 1.1e-27. These results indicate that these genes within the modules are not only highly correlated with their respective modules but also with relevant traits, further underscoring the significance of ongoing gene exploration (Figure 1B; Supplementary Figure S1C).

We then performed GO and KEGG functional enrichment analysis on the selected yellow and brown modules. The functions of receptor ligand activity, growth factor activity, and glycosaminoglycan binding, as enriched by GO, have all been demonstrated to be related to the occurrence and development of cervical cancer. Additionally, the KEGG pathways enriched by the analysis include the PI3K-Akt signaling pathway, the MAPK signaling pathway, and the Ras and Rap1 signaling pathway, all of which have also been shown to be related to the occurrence and development of cervical cancer (Figures 1C, D; Supplementary Figure S1D, S2E).

### 3.2 Identification of differentially expressed immune-related central genes in CESC samples

**FIGURE 2:**
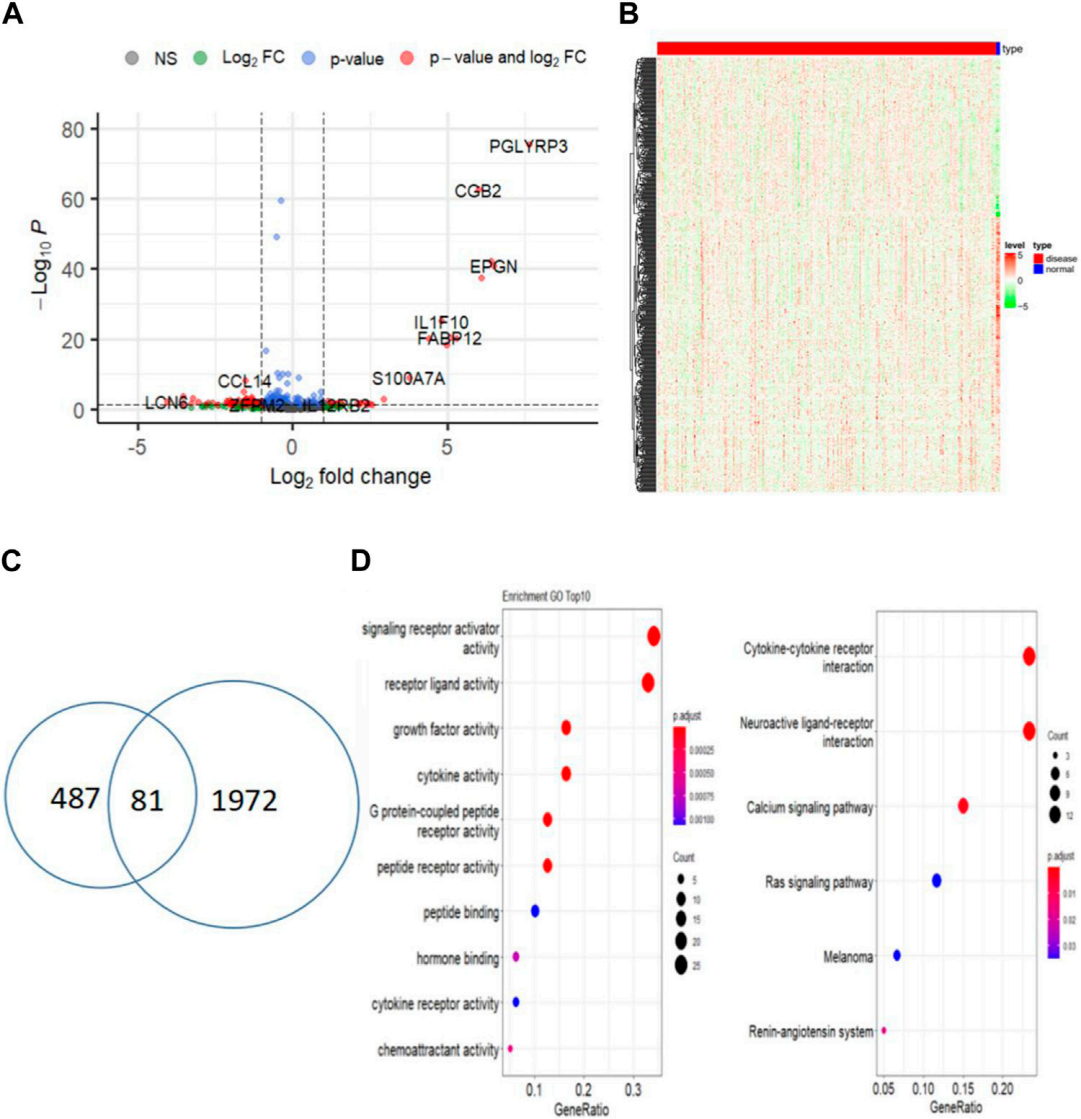
Identify differentially expressed central immune-related genes between tumor and normal samples. **(A)** Volcano plot of DEGs between CESC and normal samples; **(B)** Heatmap of differentially expressed genes in CESC (red) and normal samples (blue); **(C)** Venn diagram of immune-related DEGs in CESC and normal samples; **(D)** Functional enrichment analysis of immune-related DEGs.

Differential expression analysis of 305 cervical cancer tumors and 3 normal samples revealed 1972 significantly differentially expressed genes in cervical cancer samples (Figures 2A, B). Out of the previous 487 genes from the yellow and brown modules, 81 were identified as differentially expressed immune-related genes in cervical cancer (log2FC > 1 and *p* < 0.01) (Figure 2C). Subsequently, functional enrichment analysis was conducted, uncovering multiple functions and signaling pathways associated with cervical cancer (Figure 2D).

### 3.3 Establishment of CESC immune-related gene prognostic index (IRGPI)

**FIGURE 3:**
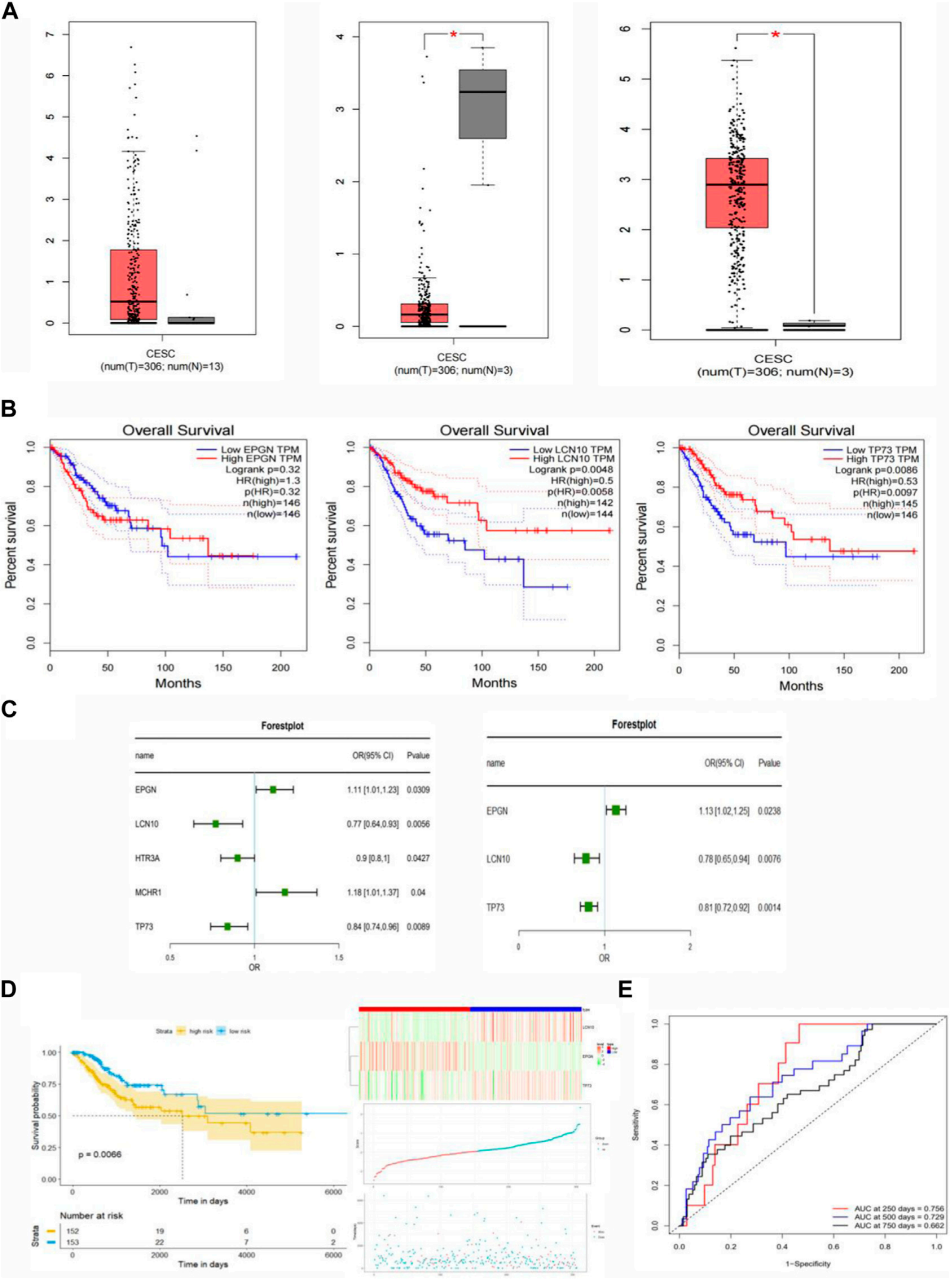
Construction of the Immune-Related Gene Prognostic Index (IRGPI) for CESC. **(A)** Differential expression of EPGN, LCN10, and TP73 genes in CESC compared to normal tissues. **(B)** Survival analysis of EPGN, LCN10, and TP73 genes. **(C)** Univariate and multivariate Cox regression analysis of survival-related genes. **(D)** Kaplan-Meier survival analysis of IRGPI scoring using TCGA data and the relationship between survival status and IRGPI score distribution in TCGA CESC cohort. **(E)** ROC curves for the 1, 3, 5, and 7-year prognostic models in CESC (TCGA cohort).

First, clinical data for patients with cervical cancer were obtained from the TCGA database. The survival status and follow-up time from this clinical data were used to establish a univariate COX proportional risk regression model for the 81 immune-related differential genes in cervical cancer that were previously identified. This helped identify genes whose expression significantly impacted the survival status of patients. Additionally, this study constructed a univariate COX proportional risk regression model for five clinical factors within the clinical data, including age, tumor stage, tumor primary, lymph node status, and distant metastasis. This allowed us to screen for clinical factors that significantly affect the survival of patients with cervical cancer (*p* < 0.05). The results revealed that five genes, namely EPGN, LCN10, HTR3A, MCHR1, TP73, and the clinical feature “STAGE,” significantly affected the survival of CESC patients. These findings indicated a significant association between these five genes and the prognosis of patients with cervical cancer (Figure 3C).

In the univariate COX regression analysis, only the relationship between each individual variable and survival status was assessed, without considering the influence of other variables. In real-world scenarios, a sample’s survival status may be jointly affected by multiple clinical factors. To address this, our study incorporated the five genes (*p* <0.05) that significantly impacted prognosis as determined by the univariate COX proportional risk regression model into a multivariate COX proportional risk regression model. Additionally, the clinical factor “Stage,” which significantly affected the prognosis of cervical cancer patients, was introduced into the model as a covariate.

In order to identify factors that independently affect the prognosis of cervical cancer, we performed a stepwise regression analysis on the candidate predictors and calculated the coefficients of the final optimal regression model. This analysis led to the identification of three genes: EPGN, LCN10, and TP73. Notably, there was no significant difference in the expression of EPGN between CESC and normal tissues, whereas the expression of LCN10 in CESC was significantly lower than in normal samples, and the expression of TP73 in CESC was significantly higher than in normal samples (Figure 3A). The results of the multivariate COX regression indicated that these three genes could be considered as prognostic markers for cervical cancer. Figure 3B displays the survival curves for EPGN, LCN10, and TP73.

To further assess the impact of these three significant immune genes on the prognosis of cervical cancer patients, we used univariate and multivariate COX proportional risk regression analyses to construct risk scores. The formula for these risk scores is as follows: Score = (EPGNexpr * 0.1181) + [LCN10expr * (−0.2517)] + [TP73expr * (−0.2069)]. Based on the calculated risk scores for each cervical cancer patient, we divided the patients into high-risk and low-risk groups using the median value as the threshold. We then used the R-package “survminer” to conduct survival analysis on cervical cancer patients in these two groups based on the risk score, and we generated KM survival curves. The results are presented in Figure 3D, where a low risk score is associated with a better prognosis, while a high risk score predicts a poor prognosis. This suggests that the three genes identified in this study are significant biomarkers for the prognosis of cervical cancer patients. To assess the performance of the prognostic model, we used ROC curves and found that the model achieved an AUC of 0.756 at 250 days, 0.729 at 500 days, and 0.662 at 750 days. These results indicate that the prognostic model, based on the risk score, can accurately predict the prognosis of cervical cancer patients (Figure 3E).

### 3.4 Validation of the predictive value of IRGPI

**FIGURE 4:**
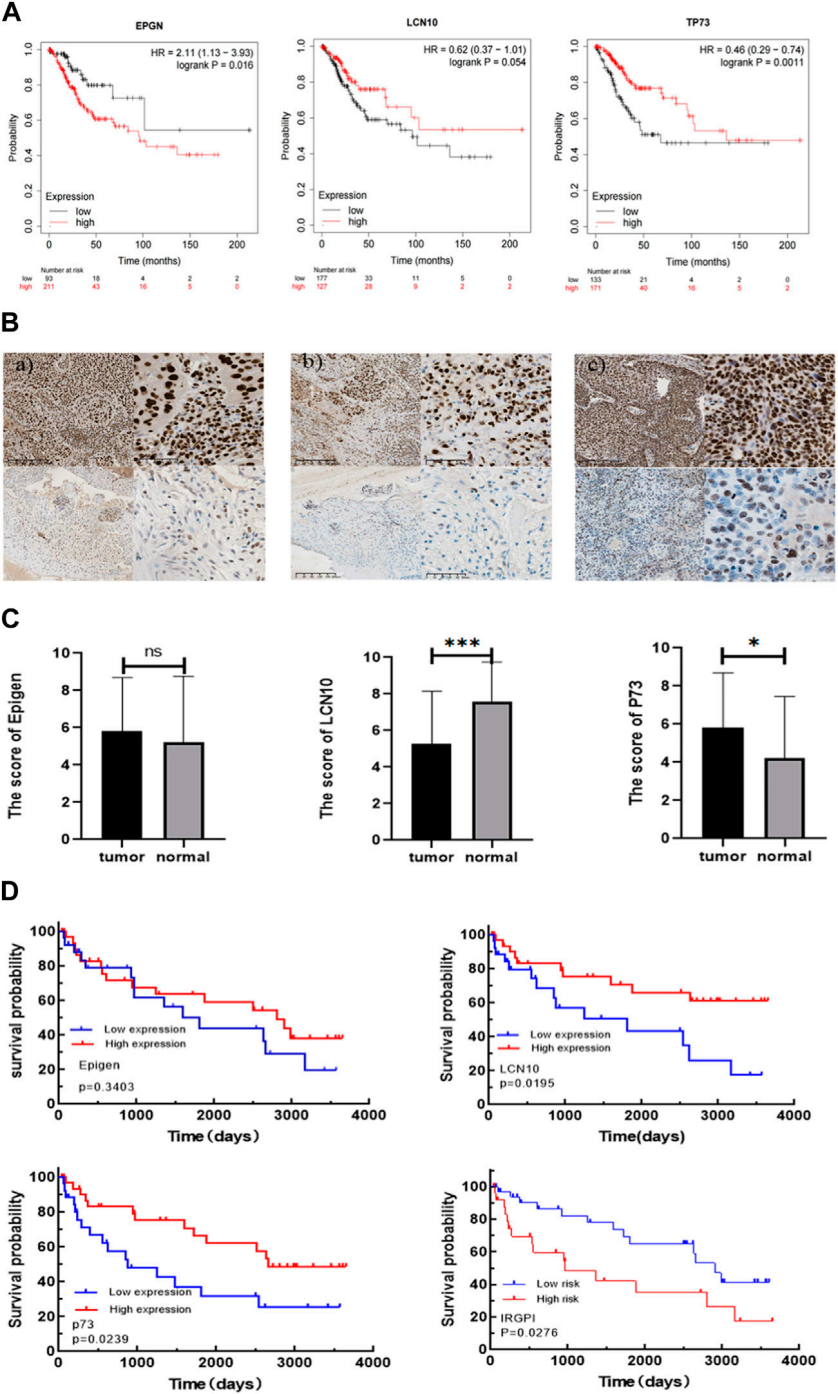
Validating the prognostic value of IRGPI. **(A)** Kaplan-Meier survival plots for the impact of EPGN, LCN10, and TP73 expression on the survival of CESC cohort patients are available in the database. **(B)** Expression of Epigen, LCN10, P73 in CESC. a) Representative samples with Epigen positive (above) and negative (below) expression; b) representative samples with LCN10 positive (above) and negative (below) expression; c) representative samples with P73 positive (above) and negative (below) expression. Scale bars: 200 μm for the left pictures in (a–c), and 50 μm for the right pictures in (a–c); **(C)** Differential expression of Epigen, LCN10, and P73 genes in CESC compared to normal cervical tissues of the patients from our hospital. **(D)** Kaplan–Meier survival analysis of Epigen , LCN10, and P73 expression and IRGPI groups using IHC scores of the patients from our hospital.

To further validate the prognostic value of EPGN, LCN10, and TP73, we conducted assessments with additional cohorts. Specifically, we utilized the CESC cohort from the Kaplan-Meier plotter database to evaluate the impact of EPGN, LCN10, and TP73 expression on overall survival. The results demonstrated that EPGN expression exhibited a significant negative correlation with the overall survival (OS) of CESC patients (log-rank test, *p* < 0.05). In contrast, LCN10 expression was positively correlated with the OS of CESC patients (log-rank test, *p* = 0.054). Similarly, the group with high TP73 expression showed better overall survival compared to the group with low TP73 expression (log-rank test, *p* < 0.05) (as depicted in Figure 4A).

We then further validated the association of IRGPI with PFS in CESC patients from our hospital. The 59 CESC patients underwent treatment after being diagnosed with cervical cancer between 2012 and 2016. Epigen (EPG) is encoded by the EPGN gene. The p73 protein is encoded by the TP73 gene. To confirm the correlation of IRGPI with the characteristics of CESC patients, we used the semi-quantitative IHC scores of Epigen, LCN10, and P73 to calculate IRGPI in the CESC patients from our hospital (Table 1). As shown in Figure 4B, Expression of Epigen, LCN10, P73 in CESC. There was no significant difference in the expression of Epigen between CESC and normal tissues, whereas the expression of LCN10 in CESC was significantly lower than in normal samples, and the expression of P73 in CESC was significantly higher than in normal samples of the patients from our hospital (Figure 4C). Consistent with the TCGA results, a high expression of LCN10 (*p* = 0.02) and P73 (*p* = 0.024) was associated with a better PFS; a low expression of Epigen (*p* = 0.34) was associated with a better PFS. Furthermore, the KM estimator showed that PFS in the IRGPI-low group was significantly longer than that in the IRGPI-high group (*p* = 0.028) (Figure 4D).

**TABLE 1:** Relationship between IRGPI and the clinicopathological characteristics of CESC patients from our hospital.

Clinical Variables	IRGPI-High(n = 26)		IRGPI-Low(n = 33)		*p* value
	n	%	n	%	
Age, years					
≤60	15	58	20	61	0.821
>60	11	42	13	39	
FIGO					
I	1	4	3	9	
II	10	38	14	42	
III	12	46	15	46	0.638
IV	2	8	1	3	
Unknown	1	4	0	0	
Pathological type					
Squamous carcinoma	23	88	32	97	
adenocarcinoma	2	8	1	3	0.367
other	1	4	0	0	
Grade					
high	9	35	8	24	
low	6	23	12	37	0.493
Unknown	11	42	13	39	
Neoadjuvant chemotherapy					
yes	8	31	11	33	0.834
no	18	69	22	67	

### 3.5 Identification of differentially expressed genes associated with IRGPI status

**FIGURE 5:**
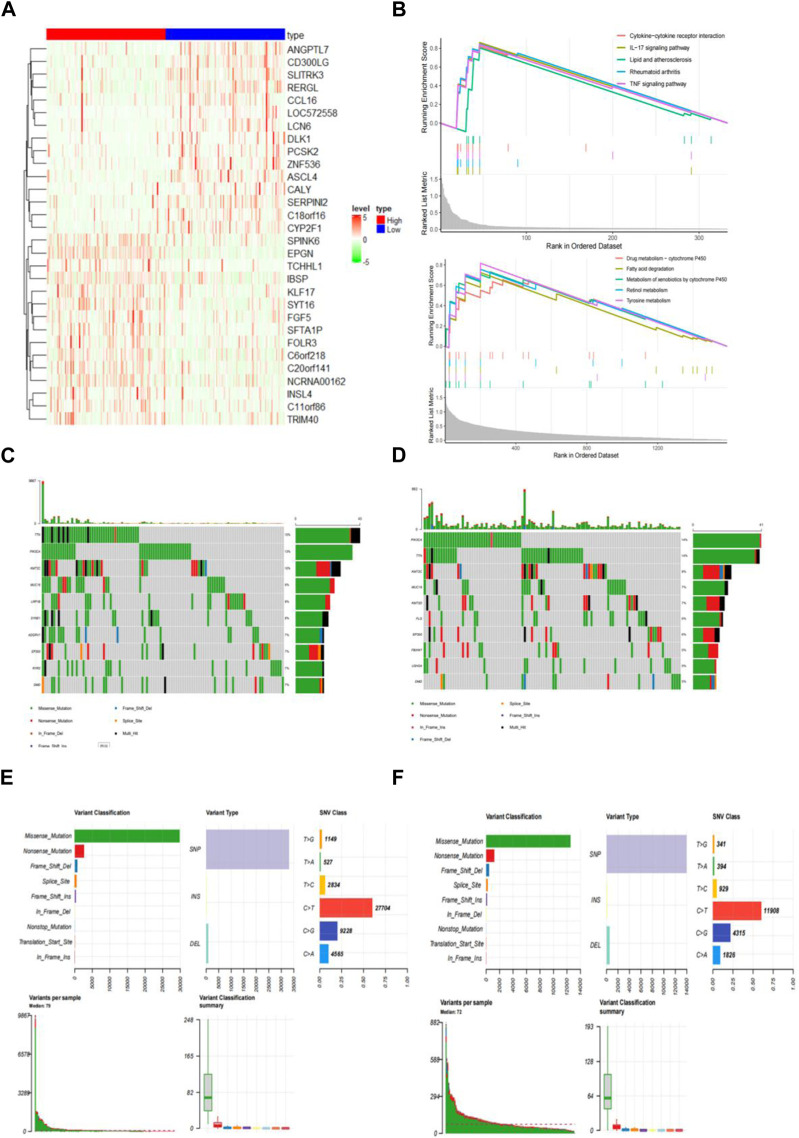
Identify differentially expressed genes (DEGs) in distinct IRGPI subgroups. **(A)** Heatmap of DEGs between high and low IRGPI risk groups. **(B)** GSEA displaying significantly enriched upregulated pathways in high (top) and low (bottom) IRGPI risk groups. **(C)** 107 (76.98%) of 139 samples with mutations in the high-risk group. **(D)** 108 (75.52%) of 143 samples with mutations in the low-risk group. **(E)** Top 10 genes with the highest mutation rates and common mutation types in the high-risk group. **(F)** Top 10 genes with the highest mutation rates and common mutation types in the low-risk group.

The 305 CESC samples were divided into high-risk and low-risk groups based on their immunization scores. Expression data from TCGA was extracted for further analysis of the high and low subgroups. Among the 2197 genes with significant differences between these groups (*p* < 0.01), 371 genes were upregulated in the high-risk group, while 1826 genes were downregulated in the same group. Heat maps were generated to visualize the top 15 genes in each group (Figure 5A).

To explore the gene sets enriched in different subpopulations, we conducted GSEA. The upregulated gene sets in the high-risk group were enriched in various immune and tumor-related pathways, including the cytokine-cytokine receptor interaction pathway and the IL-17 signaling pathway. On the other hand, the upregulated gene sets in the low-risk group were significantly enriched in pathways related to drug metabolism, such as cytochrome P450 and fatty acid degradation, among others. These pathways have also been linked to tumorigenesis and disease progression (Figure 5B).

Additionally, we performed an analysis of gene mutations to gain a deeper understanding of the immunological properties of different subpopulations. The results revealed that the mutation count in the high-risk group was significantly higher than that in the low-risk group. Missense variations were the most common mutation type, followed by nonsense deletions and frameshift deletions (Figures 5C, D). We also identified the top 10 genes with the highest mutation rates in both subpopulations (Figures 5E, F). In both groups, genes like PIK3CA, TTN, KMT2C, MUC16, EP300, and DMD exhibited high mutation rates. However, mutations in the LRP1B, SYNE1, ADGRV1, and RYR2 genes were more common in the high-risk groups. Conversely, mutations in the KMT2D, FLG, FBXW7, and USH2A genes were more prevalent in the low-risk group.

### 3.6 IRGPI correlated with the composition of immune cells in CESC samples

**FIGURE 6:**
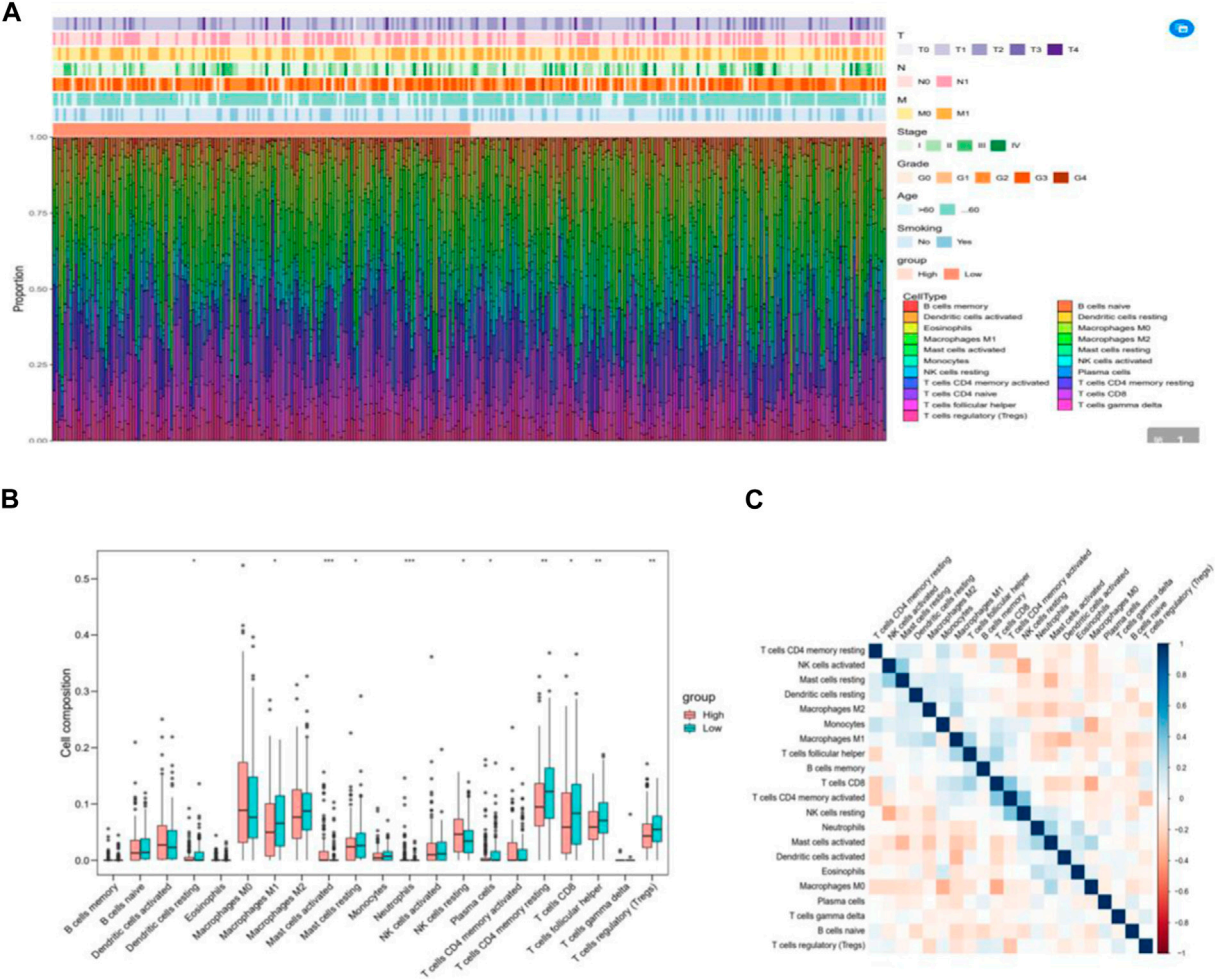
The immune cell composition in different IRGPI groups in the tumor microenvironment (TIME). **(A)** Calculation of the proportions of 21 tumor-infiltrating immune cells in individual patients using CIBERSORT. **(B)** Differences in immune cell infiltration between different IRGPI groups (pink: IRGPI high-risk group; blue: IRGPI low-risk group). **(C)** Correlations between immune cells in TNBC.

To analyze the composition of immune cells in different subgroups, we utilized CIBERSORT analysis to assess the infiltration levels of immune cells in each sample. Additionally, the Wilcoxon test was employed to compare the distribution of immune cells among various IRGPI groups (Figure 6A). Notably, we observed significant differences in the proportion of immune cells between high and low-risk groups. Upon integrating key clinical features and immunoinfiltration mapping, we discovered that the high-risk subgroup exhibited a higher abundance of activated Mast cells, Neutrophils, and resting NK cells. Conversely, resting Dendritic cells, Macrophages (M1), resting Mast cells, Plasma cells, CD4 memory resting T cells, CD8 T cells, follicular helper T cells, and T cell regulators (Tregs) were more prevalent in the low-risk subgroups (Figure 6B). Furthermore, we conducted correlation analysis for the 21 types of immune cells in cervical cancer samples. The correlations among these immune cells reveal interactions within the immune microenvironment. It is worth noting that the proportion of immune cells exhibited weak to moderate correlations (Figure 6C).

### 3.7 IRGPI is associated with T Cell rejection and dysfunction in CESC samples

**FIGURE 7:**
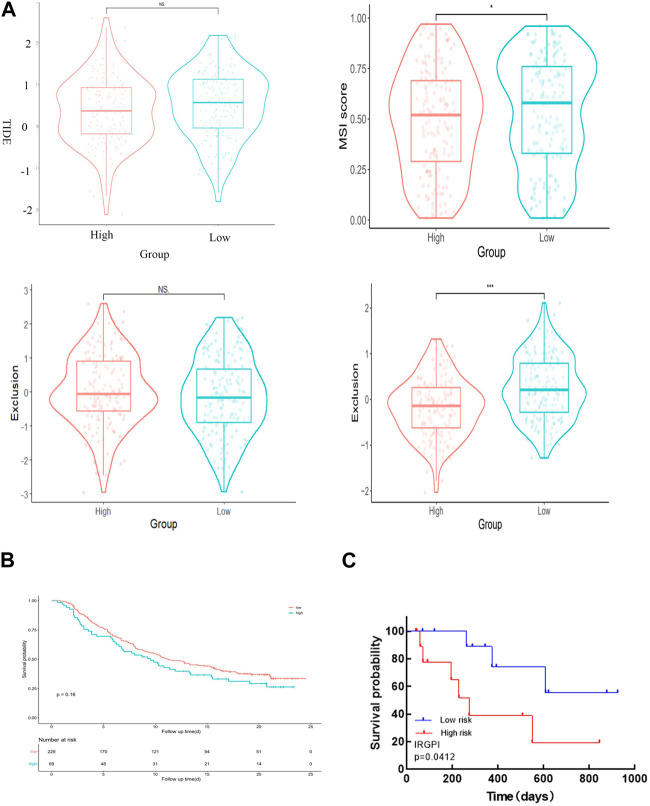
IRGPI is associated with T-cell exclusion and dysfunction in the TCGA dataset of CESC. **(A)** TIDE scores, MSI scores, T-cell exclusion scores, and T-cell dysfunction scores for different IRGPI risk groups. **(B)** The value of the score in the urothelial carcinoma cohort for anti-PD-L1 treatment. The p-value may not appear significant, but the curves can distinguish between them. **(C)** Kaplan–Meier survival analysis of PD-L1 expression and IRGPI groups using IHC scores of the patients from our hospital.

We employed TIDE to assess the potential therapeutic impact of immunotherapy in distinct subgroups. A higher TIDE predictive score indicates a greater potential for immune evasion, suggesting that patients may derive less benefit from ICI treatment. In our findings, there was no significant difference in TIDE scores between the high-risk and low-risk groups. However, the low-risk group exhibited significantly higher microsatellite instability (MSI) scores and greater T cell dysfunction compared to the high-risk group (Figure 7A). Notably, the levels of T cell rejection were relatively similar in both the low-risk and high-risk groups (Figure 7A).

### 3.8 IRGPI correlated with PD-1 and PD-L1 expression in CESC samples

**FIGURE 8:**
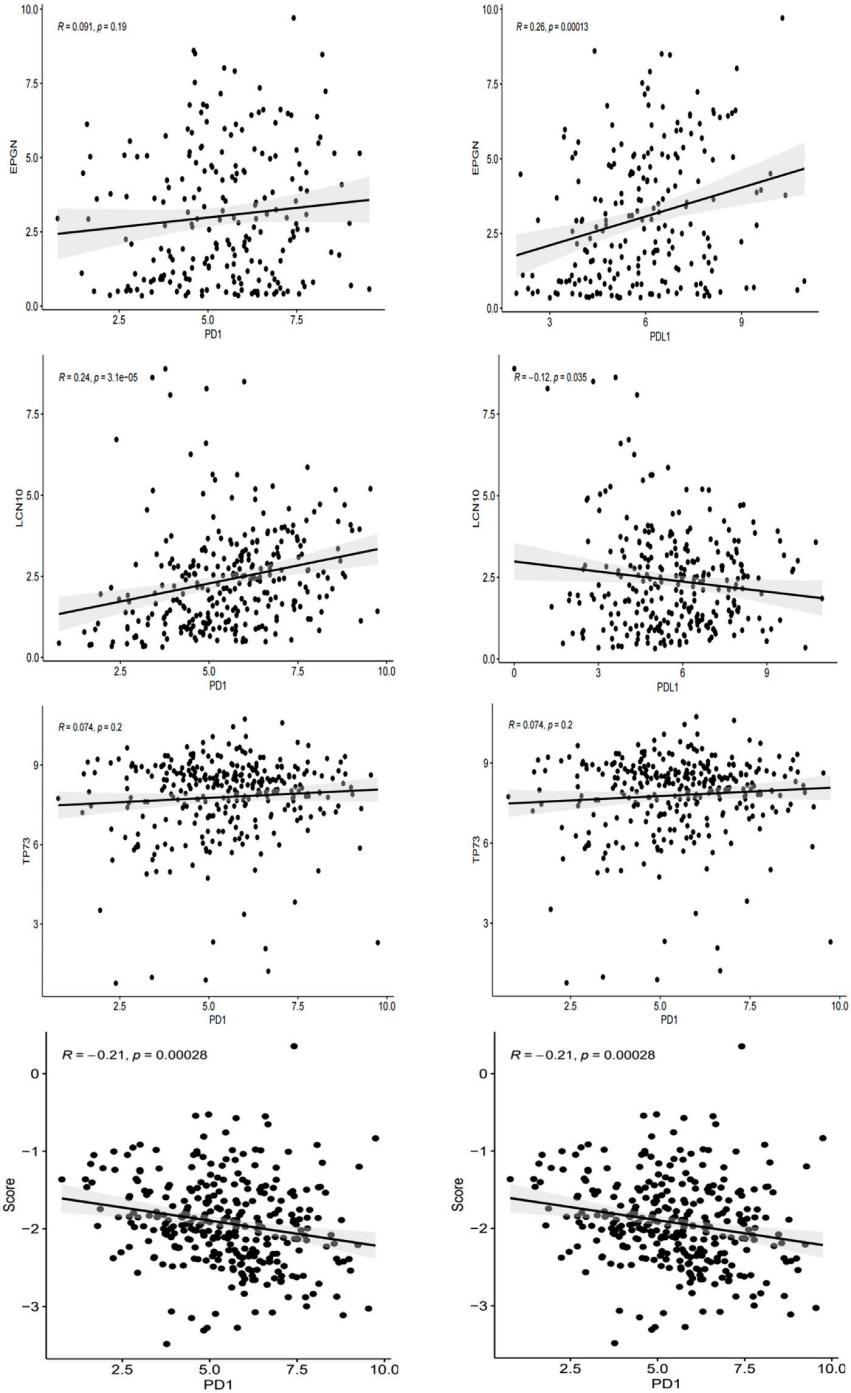
The correlation between EPGN, LCN10, TP73, IRGPI and the expression of PD-L1/PD1 in the TCGA dataset of CESC.

As PD-L1 expression is commonly used as a criterion for determining whether cancer patients should undergo ICI treatment, we examined the correlation between PD-L1/PD-1 expression levels, risk scores, and the expression of EPGN, LCN10, and TP73. Our findings revealed that EPGN exhibited a positive correlation with PD-1 and PD-L1 expression in TCGA. LCN10 showed a positive correlation with PD-1 expression but a negative correlation with PD-L1 expression. TP73 demonstrated a positive correlation with PD-1 and PD-L1 expression. Interestingly, the expression of the risk score and PD-1 and PD-L1 in the samples displayed a significant negative correlation, indicating a significant association between the risk score developed in this study and immunotherapy (Figure 8).

To validate the value of the risk score established in this study for anti-PD-L1 urothelial carcinoma, we utilized the “IMvigor210CoreBiologies” package to acquire 348 transcriptomes and corresponding clinical data from an IMvigor210-treated metastatic urothelial carcinoma cohort. Notably, the survival curve clearly shows that the samples in the high-risk group generally exhibit shorter survival times compared to those in the low-risk group, underscoring the clinical significance of the constructed risk score (Figure 7B).

To confirm the correlation of IRGPI with PD-L1 expression in CESC, we selected 23 patients with cervical cancer who had PD-L1 test reports and were treated with anti-PD-L1, and statistically analyzed the correlation between PD-L1 expression and Epigen, LCN10 and P73 expression (PD-L1 CPS score <3 was negative; CPS score >3 is positive). The results are consistent with the TCGA database, both Epigen (r = 0.542, *p* = 0.008), LCN10(r = −0.502, *p* = 0.015) and P73 (r = 0.587, *p* = 0.003) expression were correlated with PD-L1 expression (Table 2). Next, we analyzed the relationship between IRGPI and PD-L1 expression. The results show that IRGPI was negatively correlated with PD-L1 expression (r = −0.443; *p* = 0.034) (Table 2). High-risk patients had shorter survival times compared to low-risk patients, providing further evidence that IRGPI is significantly associated with immunotherapy (Figure 7C).

**TABLE 2:** Correlations between Epigen/LCN10/P73/IRGPI IHC and PD-L1 expression.

Pearson Correlation Analysis	Epigen	LCN10	P73	IRGPI IHC Score
PD-L1 expression				
R	0.542	—0.502	0.587	—0.443
p	0.008	0.015	0.003	0.034

Epigen, LCN10 and P73 expression was determined by their IHC scores. IRGPI IHC scores were Calculated with Epigen, LCN10 and P73 IHC scores. PD-L1 expression was determined by PD-L1 IHC scores in pathological reports of our hospital.

## 4 Discussion

Tumor growth and the effectiveness of ICI therapy are influenced by numerous genes, many of which are components of the TIME. To decipher the complexity of this gene network, we employed WGCNA to group immune-related genes and identify hub biomarkers within enriched modules. This led to the identification of 81 immune-related hub genes, which were further subjected to survival analysis to construct an IRGPI.

IRGPI emerged as a powerful and independent prognostic factor for Cervical Squamous Cell Carcinoma and Endocervical Adenocarcinoma patients. Its predictive ability extended across different patient cohorts, including those from TCGA and the IMvigor210 database. IRGPI forecasts improved survival for IRGPI-low patients and diminished survival for IRGPI-high patients. The consistency of these results in different cohorts underscores the considerable prognostic value of IRGPI and implies the essential role of IRGPI components in modulating TIME in CESC.

The roles of EPGN, LCN10, and TP73 in CESC are not yet well understood. EPGN is a novel ErbB ligand, and ErbB receptors belong to the tyrosine kinase receptor superfamily. They mediate the proliferation, differentiation, and survival of normal cells. ErbB family molecules are prominent candidates for therapeutic targets. Several treatment strategies targeting ErbB receptors have been used for breast cancer and lung cancer, and clinical trials are ongoing for several other malignancies. The high mRNA expression levels of unregulated ErbB ligands underscore the crucial role of ligand-receptor interactions in downstream signaling pathways (Amsellem-Ouazana et al., 2006). Novel therapeutic approaches should not only target ErbB gene products but also their ligands. In our study, we found a positive correlation between EPGN and PD-1 and PD-L1 expression, although the correlation with PD1 was not very significant. This suggests a promising prospect for combining EPGN with immunotherapy in cancer treatment, but further experimental validation and mechanistic research are needed.

Additionally, some immune system-related pathways have been found to be associated with the EPGN gene, including neutrophil degranulation, myeloid differentiation factor 88, and Toll-like receptors. Research indicates that several genes involved in immune responses may play a crucial role in promoting HPV infection (Al-Eitan et al., 2020). Prolonged high-risk HPV infection leads to cervical cancer, and we speculate that EPGN plays a key role in the development of cervical cancer. In our study, we found no significant difference in EPGN expression between CESC and normal tissues, but high EPGN expression was negatively correlated with OS in CESC patients. We have experimentally concluded that a low expression of Epigen was associated with the better survival of CESC patients in different cohorts.

Epigen (EPG) is encoded by the EPGN gene. It is a low-affinity/broad-specificity growth factor belonging to the Epidermal Growth Factor (EGF) superfamily, and it is the latest member of the mammalian EGFR ligand family. EPG mediates cell responses from survival to proliferation and migration (Yarden and Sliwkowski, 2001; Harris et al., 2003). In epithelial cells, it stimulates the phosphorylation of EGFR and downstream signaling molecules like MAPK and promotes cell proliferation in a dose-dependent manner (Strachan et al., 2001). Signaling through EGFR (ErbB1) plays a crucial role in embryonic development and adulthood (Yarden and Sliwkowski, 2001; Harris et al., 2003). Furthermore, the dysregulation of the EGFR signaling network is associated with tumor formation and invasion (Gschwind et al., 2004). Like other EGFR ligands, EPG expression is upregulated by hormones or in certain cancer types. EPG shows increased expression in human breast and prostate infiltrating adenocarcinomas (Kochupurakkal et al., 2005). EPG can induce the proliferation and differentiation of various target cell lines (Strachan et al., 2001), suggesting that EPG may be involved in tumorigenesis.

Lipocalin 10 (Lcn10) is one of the less conspicuously characterized members of the lipocalin protein family. Initially, it was identified as a gene expressed in the murine epididymis (Suzuki et al., 2004). Lcn10 deficiency promotes a pro-inflammatory polarization of macrophages by disrupting the Nr4a1 signaling pathway while inhibiting anti-inflammatory responses. Furthermore, under metabolic stress conditions, the expression of Lcn10 in macrophages is downregulated. Taken together, these findings suggest that Lcn10 may play a crucial role as an anti-inflammatory mediator in regulating macrophage function during the inflammatory process. The absence of Lcn10 may exacerbate pro-inflammatory responses in macrophages under various stress conditions while suppressing anti-inflammatory reactions. These earlier observations imply a potential role for Lcn10 as an anti-inflammatory mediator in the context of inflammation.

In the TME, tumors actively recruit macrophages in various ways. Macrophages exhibit a degree of plasticity and can be modulated by environmental stimuli. CESC cells induce macrophage polarization into M1-like tumor-associated macrophages (TAMs), which produce fewer pro-inflammatory cytokines such as tumor necrosis factor-alpha (TNF-α) and IL-10 but more anti-inflammatory cytokines like IL-92 (Sánchez-Reyes et al., 2014; Zhou et al., 2021). Tumor cells induce M2 polarization in macrophages by secreting various cytokines and growth factors. Overall, CESC cells actively recruit normal macrophages in the TME, inducing them to act as immunosuppressive TAMs. Research has shown a significant correlation between increased macrophage levels in the tumor stroma and lymphatic vessel formation and lymphatic metastasis in CESC (Li et al., 2023). The presence of TAMs in cervical cancer tumors is also associated with tumor progression and recurrence (Gorvel and Olive, 2023). Along these lines, our study results indicate that LCN10 is significantly underexpressed in CESC compared to normal samples, and LCN10 expression positively correlates with OS in CESC patients (log-rank test, *p*-value <0.05). Our research further affirms the favorable prognosis associated with Lcn10 in CESC.

It is increasingly evident that lipocalins (LCNs) play multifaceted biological roles in regulating cell proliferation, differentiation, apoptosis, and senescence (Flower, 1995; 1996; Akerstrom et al., 2000; Grzyb et al., 2006). They are also associated with the regulation of immune responses/inflammation, odor reception, reproduction, cancer development, and metabolic disorders, as well as cardiovascular remodeling (Virtanen, 2021; Ganfornina et al., 2022; Redl and Habeler, 2022). Our study establishes a positive correlation between LCN10 and PD-1 expression while showing a negative correlation with PD-L1 expression, further underscoring the connection of LCN10 with immune responses. As carrier proteins, LCNs play a role in the general clearance of hydrophobic molecules both intracellularly and extracellularly (Flower, 1996). Clinically, LCNs have been widely explored as biochemical biomarkers for diagnosing human diseases (Bacci et al., 2015; Zabetian-Targhi et al., 2015; Bergwik et al., 2021; Sawyer, 2021; Steinhoff et al., 2021; Angelika et al., 2023). Studies have observed a significant increase in LCN2 expression in macrophages under metabolic stress conditions, while LCN10 expression is significantly downregulated (Li et al., 2022). Future research is needed to clarify whether increased LCN10 expression/activity in macrophages or direct administration of recombinant LCN10 protein has therapeutic potential in cervical cancer. Furthermore, exploring the relevance of other lipocalin family members to cervical cancer development is necessary.

TP73 is a tumor suppressor, a member of the p53 gene family, highly homologous to TP53, and plays a unique role in neurodevelopment and apoptotic responses to DNA damage (Stella et al., 2019). It has a pivotal regulatory function in various processes, including embryonic development, tissue homeostasis, and cancer. TP73 has garnered significant attention in the field of cancer management, mainly because it can mimic and/or substitute for the anti-cancer function of p53. Unlike p53, TP73 rarely mutates in cancer, making it less susceptible to intratumoral and intertumoral mutational heterogeneity (p73-Governed miRNA Networks: Translating Bioinformatics Approaches to Therapeutic Solutions for Cancer Metastasis—PubMed, n. d.). Reports indicate that TP73 has roles in cell cycle arrest, apoptosis, and genome stability in multiple cancers (Candi et al., 2014). Various TP73 variants have been identified to be highly expressed in several cancers (Yao et al., 2019). Altered TP73 expression is observed in most human cancers and is associated with adverse outcomes in colorectal cancer patients. Studies on colorectal cancer have found that TP73-positive tumor patients have a greater malignancy risk and shorter survival (Kotulak et al., 2016; Chu et al., 2020). Our study results show that TP73 is significantly overexpressed in CESC compared to normal samples, consistent with existing literature. The group with high TP73 expression exhibits better OS than the low TP73 expression group (log-rank test, *p* = 0.0086). The differences in survival analysis between our study and the literature may be related to the consideration of different TP73 variants. TP73 can be translated into numerous isoforms with both oncogenic and tumor-suppressive functions and participates in complex signaling cascades with TP53 and TP63 (Rodríguez et al., 2018).

The p73 protein is a major member of the p53 protein family encoded by the TP73 gene. It is now recognized that p73 not only impacts numerous cancer-related pathways but also regulates various processes of embryonic development and tissue homeostasis. Studies have revealed that TP73+/− mice display a heightened susceptibility to cancer, underscoring the undeniable association between p73 and cancer (Flores et al., 2005). In fact, there is a degree of functional overlap between p53 and p73, and p73 can inhibit cancer cell growth independently of p53 (Jost et al., 1997; Collavin et al., 2010; Ozaki et al., 2010). There are studies that show the p73 activators may have a different anti-cancer effect in non-aggregative and aggregative p53 mutants (Cai et al., 2022).

P73 isoforms are crucial for the normal functioning of the immune system. In the context of cancer, the TAp73 subtype is considered to have anti-cancer effects (Stiewe and Pützer, 2000; Malik et al., 2021). On one hand, resolution of macrophage-mediated innate immunity and inflammatory responses requires TAp73. TAp73 KO alters macrophage polarization, extending the maintenance of the M1 effector phenotype at the expense of the M2 phenotype, thereby impairing the resolution of inflammation (Tomasini et al., 2013). Studies have shown that the overexpressed tumor suppressor PCBP1 favored the production of long isoforms of p73 in human cervical carcinoma cells, thereby inducing upregulated ratio of Bax/Bcl-2, the release of cytochrome c and the expression of caspase-3 (Chen et al., 2022). On the other hand, p73 is vital for the normal functioning of the immune system. In adaptive immunity, Ren et al. (Ren et al., 2020) recently discovered that p73 acts as a negative regulator of Th1 immune response by suppressing IFN γ transcription and downregulating IFN γ production. Our study reveals a positive correlation between TP73 and PD-1 and PD-L1 expression. TP73 rarely mutates in tumors, making p73 an attractive therapeutic target, especially for cancers with ineffective or disrupted p53 pathways (Dötsch et al., 2010). Therefore, gaining a deeper understanding of the p73 pathway is of significant importance for advancing new cancer treatment strategies. Investigating combined immunotherapy targeting TP73 is also a worthy avenue for further exploration.

Due to the pivotal roles of EPGN, LCN10, and TP73 in the TIME, we conducted an analysis of immune cell profiles in CESC to investigate the connection between IRGPI and the composition of tumor-infiltrating immune cells. The distribution of immune cell types varied between the two IRGPI subgroups. Dendritic cells resting, Macrophages M1, Mast cells resting, Plasma cells, T cells CD4 memory resting, CD8 T cells, T cells follicular helper, and Tregs were more abundant in the IRGPI-low subgroup, while Mast cells activated, Neutrophils, and resting NK cells were more prevalent in the IRGPI-high subgroup. Numerous studies have demonstrated that dense infiltration of T cells, particularly cytotoxic CD8 T cells, is indicative of a favorable prognosis (Bindea et al., 2013; Gentles et al., 2015; Fridman et al., 2017), consistent with previous clinical observations in CESC (Piersma et al., 2007). In most tumor types, Neutrophils have been shown to promote tumor development. Neutrophils within tumors typically contribute to immunosuppression and are associated with a poor prognosis. Conversely, M1 macrophages are known for their anti-tumor activity by promoting immune responses, thus predicting a favorable prognosis in various cancer types (Solinas et al., 2009; Qian and Pollard, 2010; Ruffell and Coussens, 2015; Fridman et al., 2017; Metzemaekers et al., 2023). These findings suggest that the IRGPI-low subgroup has a more favorable immune microenvironment than the IRGPI-high subgroup.

Recently, a novel algorithm called TIDE was developed to model tumor-immune evasion by evaluating the extent of T cell exclusion and the priming of infiltrating cytotoxic T lymphocytes (CTLs) (Jiang et al., 2014). TIDE has shown superior performance in assessing the effectiveness of first-line ICI therapy in melanoma patients compared to widely used ICI therapy biomarkers, such as tumor mutation burden and PD-L1 expression. A higher TIDE predictive score reflects a greater potential for immune escape, suggesting that patients are less likely to benefit from ICI treatment. In our results, MSI scores and T cell dysfunction were significantly higher in the low-risk group than in the high-risk group.

Next, we investigated the relationship between PD-1/PD-L1 expression and IRGPI. Given that PD-L1 expression is the most commonly used criterion for determining whether patients should receive ICI treatment, we examined the correlation between PD-L1/PD1 expression levels and the risk score. In the TCGA dataset, we observed that IRGPI was inversely correlated with both PD-1 and PD-L1 expression. Furthermore, the expression of PD-1 and PD-L1 positively correlated with EPGN and TP73 expression, while LCN10 expression was positively associated with PD-1 expression and negatively associated with PD-L1 expression. These results suggest a significant correlation between the risk score established in this study and immunotherapy potential.

Although we consistently observed a positive correlation between PD-L1 and EPGN or TP73 expression, the underlying molecular mechanisms behind these observations remain unclear. In Epstein-Barr virus-associated gastric cancer (EBVaGCs), TP73 methylation is detected in 92%–100% of cases, compared to just 5% in EBV-negative gastric cancer (Chang et al., 2006; Ushiku et al., 2007). Small RNA encoded by the Epstein-Barr virus (EBER) expression and loss of the EBV genome were associated with a decrease in the number of PD-L1-positive immune cells and CD8^+^ tumor-infiltrating T lymphocytes. One possible explanation for this phenomenon is that tumors can evade immune surveillance by down-regulating or reducing virus-associated antigens, which may benefit tumor progression (Kondo et al., 2023). However, there are no reports regarding the regulatory relationship between EPGN and PD-L1. Nevertheless, IRGPI is a valuable and reproducible measure for assessing PD-L1 expression in CESC.

## 5 Conclusion

In conclusion, the study unveiled a simplified IRGPI consisting of three key genes. IRGPI serves as an excellent prognostic indicator for CESC patients. It not only reflects the immune cell composition within the tumor-immune microenvironment but also indicates the status of TILs, as well as PD-1 and PD-L1 expression. Patients with low IRGPI values may benefit more from CTL activation in ICI therapy. Further research should focus on understanding the molecular mechanisms underlying the relationships between EPGN, LCN10, TP73, and ICI therapy, and explore their therapeutic potential in cervical cancer treatment.

## Data Availability

The original contributions presented in the study are included in the article/Supplementary Material, further inquiries can be directed to the corresponding author.
