# Supplementary figures and images for: Decoding the immune landscape: a comprehensive analysis of immune-associated biomarkers in cervical carcinoma and their implications for immunotherapy strategies
Source: Front Genet. 2024 Jun 12;15:1340569. doi: 10.3389/fgene.2024.1340569 (PMC11199791; doi:10.3389/fgene.2024.1340569)

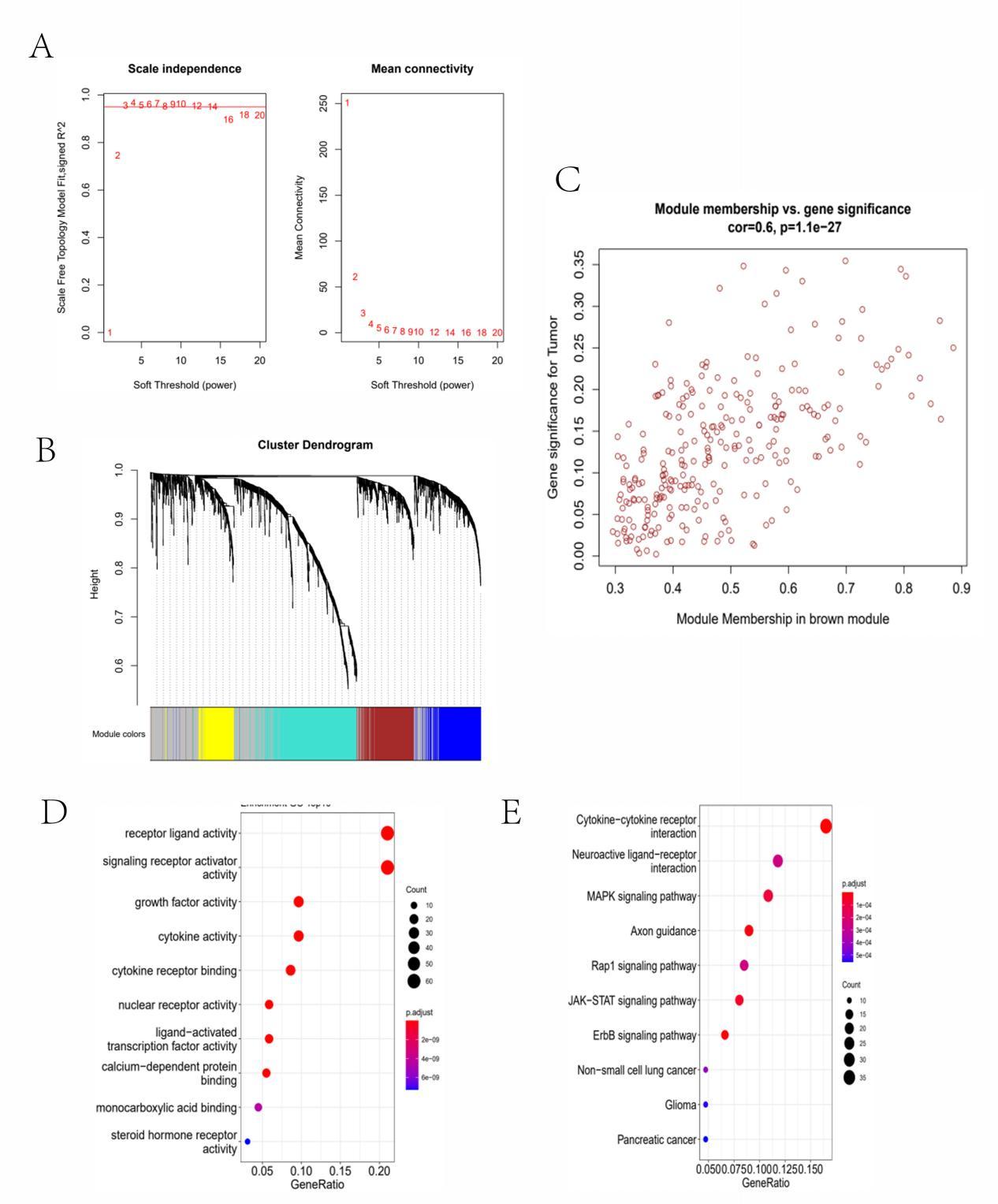

Supplement: Supplementary file 1 [file Image1.JPEG]

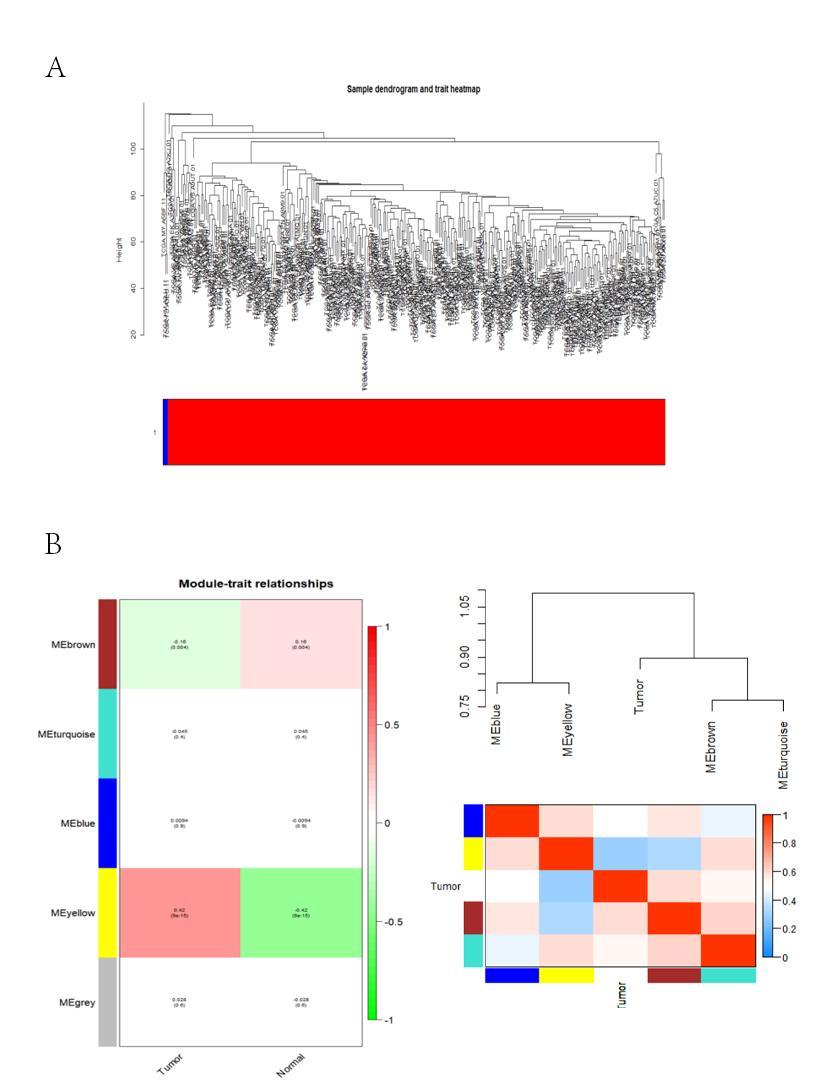

Supplement: Supplementary file 2 [file Image2.JPEG]
